# *Salvia fruticosa* Induces Vasorelaxation In Rat Isolated Thoracic Aorta: Role of the PI3K/Akt/eNOS/NO/cGMP Signaling Pathway

**DOI:** 10.1038/s41598-017-00790-9

**Published:** 2017-04-06

**Authors:** M. Akhtar Anwar, Ali A. Samaha, Samar Ballan, Alaaeldin I. Saleh, Rabah Iratni, Ali H. Eid

**Affiliations:** 1 College of Arts and Sciences, Qatar University, Department of Biological and Environmental Sciences, Doha, P.O. Box 2713 Qatar; 2 Lebanese International University, Department of Biomedical Sciences, Beirut, P.O. Box 146404 Lebanon; 3 Lebanese University, Faculty of Public Health IV, Department of Anatomy, Zahlé, Lebanon; 4 College of Science, United Arab Emirates University, Department of Biology, Al Ain, P.O. Box 15551 United Arab Emirates; 5 American University of Beirut, Faculty of Medicine, Department of Pharmacology and Toxicology, Beirut, P.O. Box 11-0236 Lebanon

## Abstract

*Salvia fruticosa* (SF) Mill. is traditionally used for its antihypertensive actions. However, little is known about its pharmacologic and molecular mechanisms of action. Here we determined the effects of an ethanolic extract of SF leaves on rings of isolated thoracic aorta from Sprague-Dawley rats. Our results show that SF extract increased nitric oxide production and relaxed endothelium-intact rings in a dose-dependent (0.3 µg/ml–1 mg/ml) manner, and the maximum arterial relaxation (R_max_) was significantly reduced with endothelium denudation. Pretreatment of endothelium-intact rings with L-NAME (a non-selective inhibitor of nitric oxide synthase, 100 µM), or ODQ (an inhibitor of soluble guanylyl cyclase, 10 µM) significantly diminished SF-mediated vasorelaxation. Furthermore, SF induced Akt phosphorylation as well as increased cGMP levels in rings treated with increasing doses of SF. Prior exposure to PI3K inhibitors, wortmannin (0.1 µM) or LY294002 (10 µM), decreased cGMP accumulation and attenuated the SF-induced vasorelaxation by approximately 50% (R_max_). SF-evoked relaxation was not affected by indomethacin, verapamil, glibenclamide, tetraethylammonium, pyrilamine or atropine. Taken together, our results indicate that SF induces endothelium-dependent vasorelaxation through the PI3K/Akt/eNOS/NO/sGC/cGMP signaling pathway. Our data illustrate the health-orientated benefits of consuming SF which may act as an antihypertensive agent to reduce the burden of cardiovascular complications.

## Introduction

Cardiovascular disease (CVD) remains the leading cause of death in the world^1^. Along with many other risk factors, hypertension continues to be a major contributor to this mortality. Not only does hypertension kill one in every eight people, but it also threatens as many as 1 billion people worldwide^2^. Despite the tremendous therapeutic advances made in recent decades, current cardiovascular drugs remain inefficient at treating a significant proportion of patients^3^. Therefore, there is an increasing need for other approaches that could provide new avenues to combat CVD. Especially during the last 10 years, herbal medicine has emerged as a significant alternative for the treatment of several diseases including CVD^4–6^.

Herbs and other medicinal plants have been at the foundation of drug development from the very inception of global pharmaceutical industry, and continue to attract focus of attention for research, worldwide^7, 8^. Moreover, the public from both developed and developing nations hanker for alternative, cheaper and safer drugs, which may be used for prolonged duration with minimal side-effects^7^. Our knowledge regarding the beneficial constituents of plants, particularly related to ethnomedicine and ethnobotanicals, remains at the stage of infancy. However, the present interest in herbal medicine will certainly lead to an expansion in newer classes of botanical-based drugs during the next decade or thereafter. This action is urgently required, as many of the currently available drugs are not without serious undesired side effects^3^. Moreover, herbal remedies and their constituents are associated with amelioration of a number of global endemics linked to high morbidities and mortalities, including cardiovascular disease^5, 6, 9, 10^, metabolic syndrome^11, 12^, cancer^13–16^ and neurodegenerative diseases^17–19^.

There are a multitudinous number of medicinal herbs belonging to the genus Salvia (sage). Indeed, sage has a worldwide distribution with approximately 1000 species, and is the largest genus in the family Lamiaceae. Several species of Salvia have demonstrable physiological and pharmacological attributes associated with improvement and prevention in vascular dysfunction, including blood pressure-lowering effects^20–24^. Interestingly, culinary herbs such as sages are important components of diet in the Mediterranean basin, where the demographics of cardiovascular-associated morbidity and mortality is low^25^.

**Figure 1:**
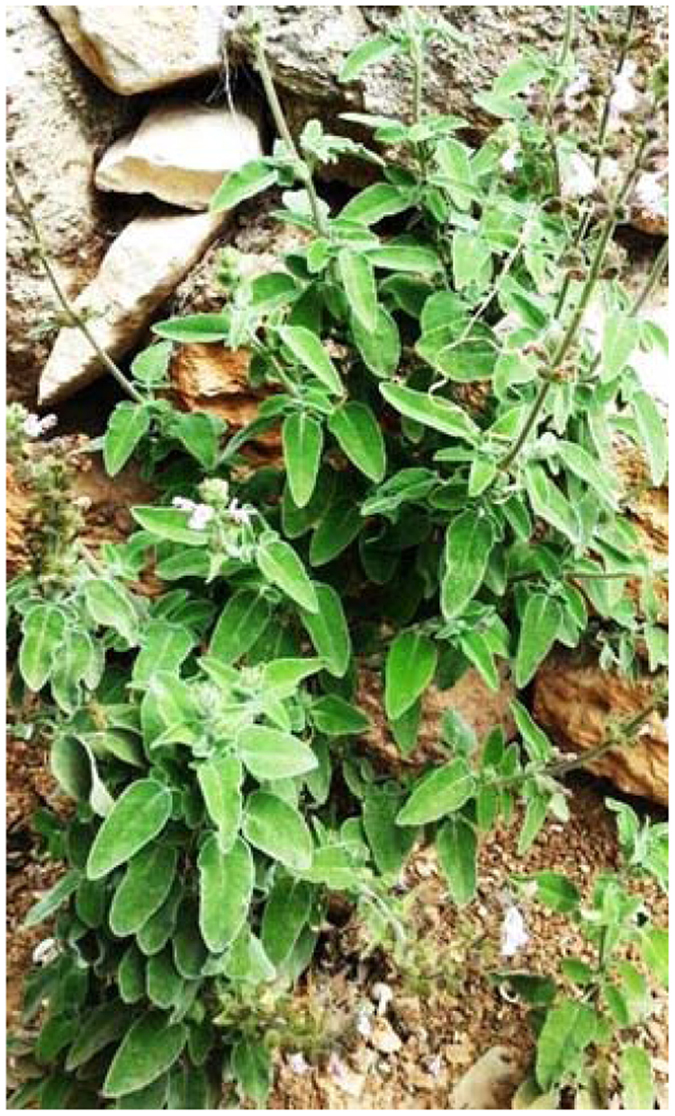
*Salvia fruticosa* Mill. (Sage). A photograph showing the aerial parts of SF. For medicinal uses, leaves are the most commonly consumed part of this plant.

*Salvia fruticosa* Mill. (Fig. 1) (also referred to as *S. libanotica* Boiss. & Gaill., *S. triloba* L.f., and *S. cyrpia* Unger & Kotschy) is commonly known as the East Mediterranean sage and is widely used in the gastronomy of the Levant^26^. It is a perennial herb with trifoliate hairy leaves that are grey to green in color. Its flowers are lavender-pinkish in color and are held in a reddish five-pointed hairy calyx^27^. Accumulating evidence reveals a remarkable array of therapeutic properties for this herb. In addition to its many beneficial biological activities in its arsenal, sage is also endowed with anti-inflammatory^28^, anti-oxidant^29, 30^ and anti-proliferative^31^ effects, as well as the inhibition of smooth muscle contraction^32^.

Traditionally, as part of the armamentarium of ethnomedicine of the Eastern Mediterranean basin and the hinterland beyond, leaves of this herb have been used for their anti-hypertensive effects^26, 33^. An ethno-botanical study has divulged the ethno-pharmaceutical use of *Salvia fruticosa* Mill. by British Turkish-speaking Cypriots residing in London (United Kingdom) for amelioration of high blood pressure (BP)^34^. Furthermore, in Cyprus, the aerial parts of this sage are commonly used for its hypotensive effects^35, 36^. All of these remedial homeostatic effects are bestowed by a rich and diverse population of phytochemicals. The principle quantitative components of polyphenols isolated from SF are: hydroxycinnamic acid derivatives: rosmarinic acid (caffeic acid dimer), salvianolic acids (caffeic acid polymers), caffeic acid phenethyl ester (caffeic acid derivative); phenolic diterpenes: carnosic acid and carnosol; and flavonoids: luteolin-7-O-glucoside and rutin^37, 38^. In framework of the present study, rosmarinic acid exerts an arterio-relaxant effect in rat isolated thoracic aorta^39^. Moreover, rosmarinic acid was reported to lower BP in fructose-fed hypertensive rats. The drop in BP arose through a mechanism entailing a fall in circulating levels of endothelin-1, suppression in angiotensin-converting enzyme activity, and augmentation of nitric oxide synthesis^40^.

Despite all these common uses, the mechanism by which this medicinal herb elicits its therapeutic effects on the vasculature remains unknown. This study was therefore undertaken to investigate the vasorelaxant function of SF in the rat aorta, and to establish the underlying pharmaco-biochemical mechanism of action.

## Results

### Effect of SF on relaxation of rat isolated aortic rings

**Figure 2:**
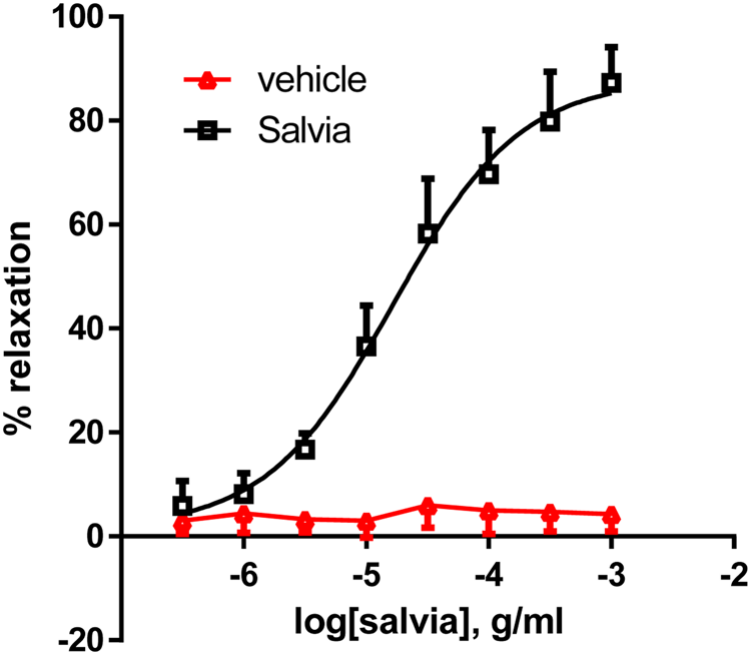
Effect of SF extract on vasorelaxation of aortic rings. Cumulative dose-dependent curves for SF-induced relaxation and of the vehicle (ethanol) in rat aortic rings. n = 7.

First, we determined the vasorelaxing effect of SF on endothelium-intact rat aortic rings. Figure 2 provides definitive evidence for SF-induced, dose-dependent relaxation of rat isolated aortic rings pre-contracted with norepinephrine, yielding a pED_50_ value of 4.78 ± 0.08 g/ml with 95% confidence interval: 4.93–4.63 g/ml, whereas the R_max_ is 88 ± 4%. The highest percentage volume of vehicle (ethanol) used did not cause a significant relaxation effect on rat aortic ring.

### Role of the endothelium in SF-induced relaxation

**Figure 3:**
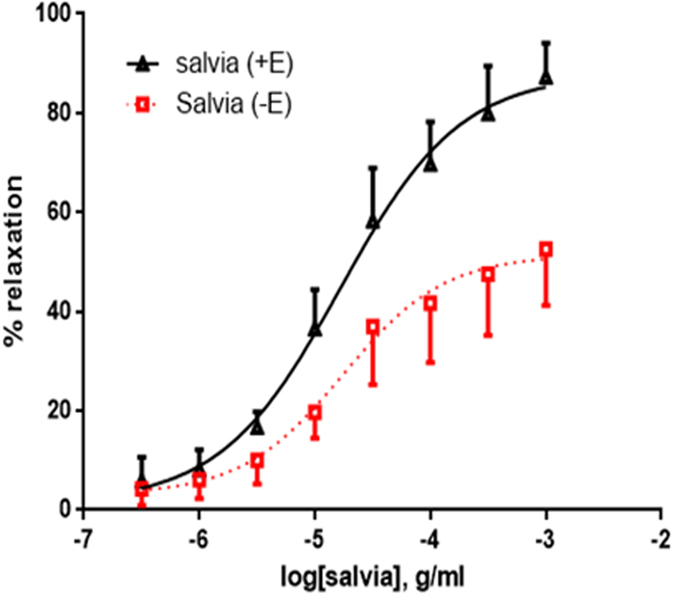
Role of endothelium in SF-induced relaxation. Cumulative dose-response curves for SF in isolated norepinephrine-precontracted rat aortic rings either with intact (+E; triangles) or denuded endothelium (−E; squares). n = 7; *p* < 0.01 for +E versus −E.

In this series of experiments, we examined the vasorelaxing effect of SF on vascular smooth muscle using endothelium-denuded rat aortic rings. As illustrated in Fig. 3, SF induced relaxation in a dose-dependent manner for endothelium-intact and denuded vessels; however, removal of the endothelium causes a significant decrease (*p* < 0.01) in the vasorelaxation produced by SF from a dose of 10 µg/ml to 1 mg/ml. The relevant parameter values are: pED_50_ = 4.78 ± 0.08 g/ml with 95% confidence interval: 4.93–4.63 g/ml (with endothelium), 4.76 ± 0.13 with 95% confidence interval: 5.02–4.50 g/ml (without endothelium). Similarly, R_max_ is 88 ± 4% for endothelium-dependent relaxation and 51 ± 3% for endothelium independent relaxation (*p* < 0.01). It is important to note that while endothelium-dependent relaxation is obvious, endothelial denudation did not seem to abolish the vasorelaxant effect completely. This suggests that there could be an endothelium-independent effect of SF.

### Aortic responses to SF in the absence and presence of L-NAME or ODQ

**Figure 4:**
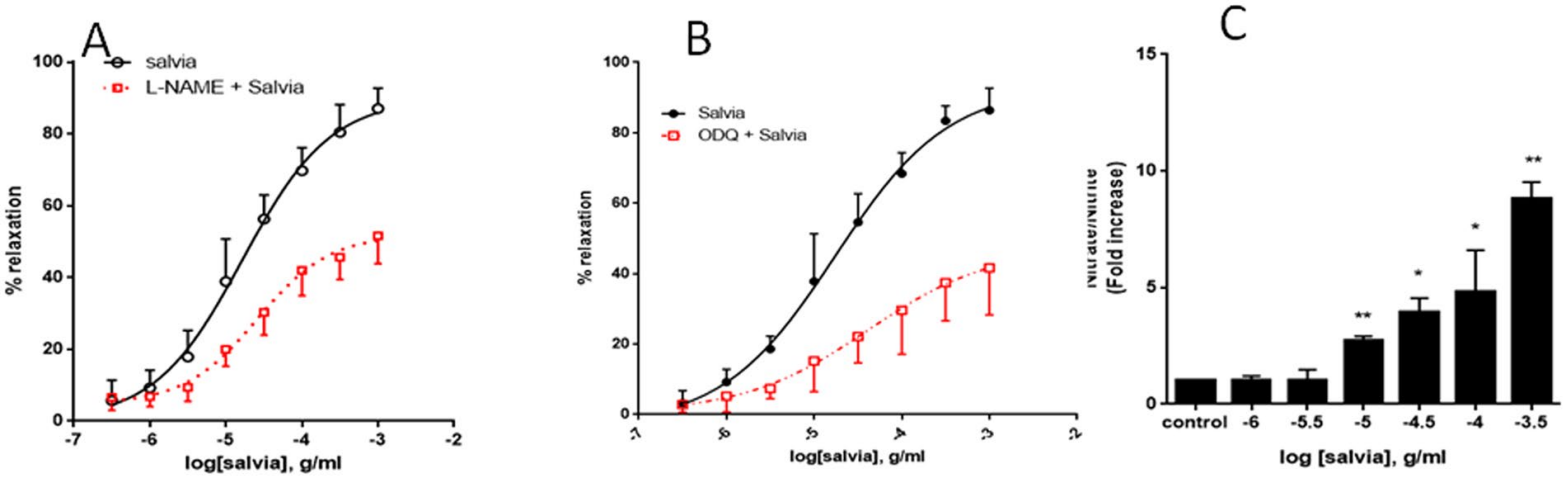
Effect of L-NAME or ODQ on SF-induced relaxation. (**A**) Endothelium-intact rings were incubated with cumulative doses of SF in the absence (circles) or presence of L-NAME (100 µM; squares. Data represent mean ± SEM (n = 7; *p* < 0.01 for Salvia versus L-Name plus Salvia). (**B**) Endothelium-intact rings were incubated with cumulative doses of SF in the absence (circles) or presence of ODQ (1 µM; squares). n = 7; *p* < 0.01 for Salvia versus ODQ plus Salvia. (**C**) Endothelium-intact rings were incubated with increasing doses of SF and levels of NO determined. n = 3; *denotes a *p* < 0.05 and **a *p* < 0.01 (compared to control).

Next, we determined the role of nitric oxide and soluble guanylate cyclase in aortic relaxation responses to SF. The SF-induced vasorelaxation was significantly attenuated by the presence of L-NAME, an inhibitor of endothelial nitric oxide synthase (Fig. 4A). This confirms a role for NO in SF-induced aortic relaxation. The pED_50_ values are 4.72 ± 0.07 g/ml with 95% confidence interval: 4.86–4.58 g/ml (without L-NAME), 4.59 ± 0.09 with 95% confidence interval: 4.78–4.40 g/ml (with L-NAME). Similarly R_max_ is 90 ± 4% prior to L-NAME exposure and 52 ± 3% in the presence of L-NAME (*p* < 0.01).

In the presence of ODQ (sGC inhibitor, Fig. 4B), the SF-dependent vasorelaxation was significantly inhibited by approximately 50%, and there was no change in potency. The pED_50_ values = 4.76 ± 0.10 g/ml with 95% confidence interval: 4.97–4.55 g/ml (no ODQ exposure) and 4.38 ± 0.37 with 95% confidence interval: 5.14–3.63 g/ml (with ODQ) to increasing doses of SF-mediated relaxation. Similarly R_max_ is 93 ± 6% for ODQ-independent relaxation and 48 ± 12% for ODQ-dependent relaxation (*p* < 0.01).

To further confirm the involvement of NO, rings were incubated with increasing doses of SF and the level of NO determined. As shown in Fig. 4C, SF caused a significant and dose-dependent increase in the production of nitrate/nitrite, indicative of increased NO production. This suggests that SF elicits its effects via increasing NO levels.

### Effect of SF on production of cGMP in aortic rings

**Figure 5:**
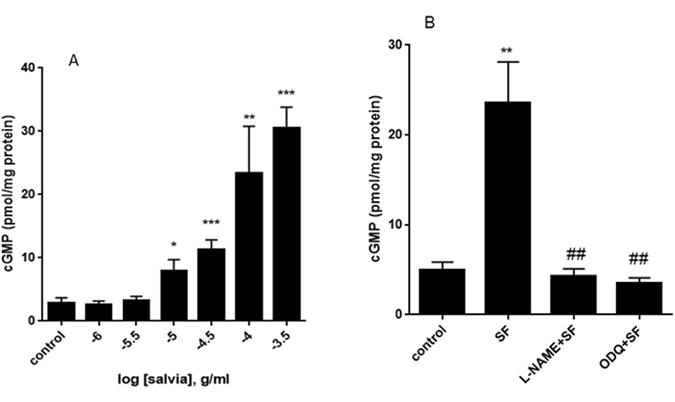
Modulation of cGMP levels by SF, L-NAME and ODQ. (**A**) Rings were incubated in the absence (control) or presence of increasing concentrations of SF. cGMP immunoassay followed and levels of cGMP were determined. n = 6; *denotes a *p* < 0.05, **a *p* < 0.01 and ***a *p* < 0.001 compared to control. (**B**) Rings were pretreated for 30 minutes with L-NAME (100 µM) or ODQ (1 µM) followed by SF (0.67 mg/ml) and cGMP levels determined. n = 5; **denotes a *p* < 0.01 compared to control; ^##^denotes a *p* < 0.01 compared to SF).

Since SF-induced vasorelaxation was significantly decreased by L-NAME and ODQ, these results suggested we address the question if SF modulates the level of cGMP. This nucleotide monophosphate is released from GTP by soluble guanylyl cyclase when nitric oxide docks onto its binding domain on the enzyme. Treatment with cumulative doses of SF evoked a significant and dose-dependent sigmoidal increase in the production of cGMP (pED_50_: 4.26 ± 0.14 g/ml; Rmax: 33 ± 5 pmol/mg protein) (Fig. 5A). In vehicle-treated rings, the cGMP content was 2.82 ± 0.87 (mean ± SEM) pmole/mg protein. However, in rings treated with 0.3 mg/ml SF, the cGMP level was 30.50 ± 3.31 (mean ± SEM) pmole/mg protein (*p* < 0.001).

To confirm if this SF-increased cGMP accumulation is mediated by eNOS and/or sGC, rings were pre-treated with either L-NAME (100 µM) or ODQ (1 µM) followed by SF. Indeed, pretreatment with either inhibitor significantly inhibited the SF-induced cGMP accumulation (Fig. 5B). Our results also show that 8-(4-Chlorophenylthio)-guanosine 3′,5′-cyclic monophosphate, a potent and selective activator of cGMP dependent protein kinase (PKG), evokes relaxation of aortic rings (data not shown).

### Involvement of phosphoinositide 3-kinase(PI3K)/Akt (protein kinase B) signaling pathway axis in the SF-induced relaxation of aortic rings

**Figure 6:**
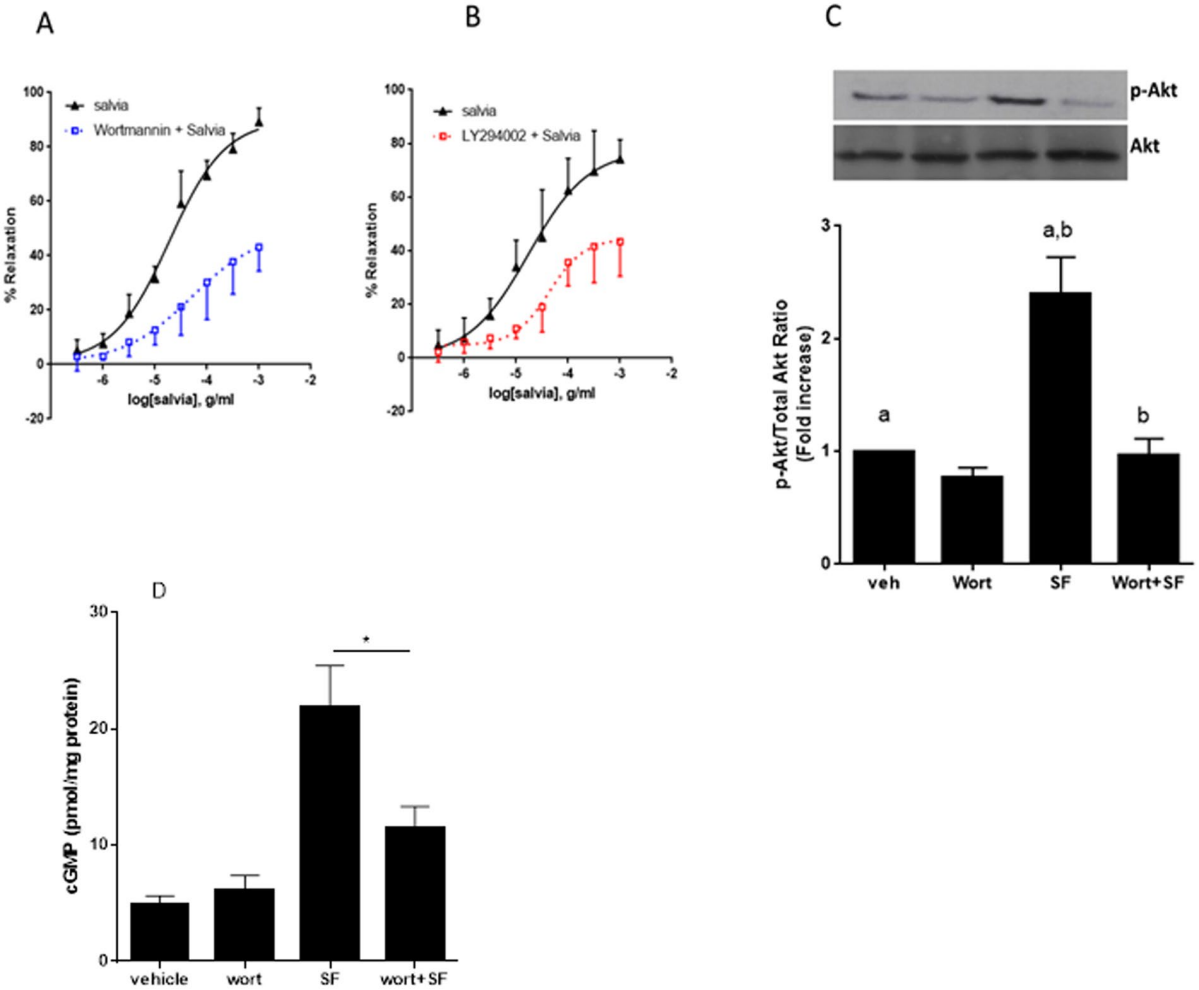
Interplay between SF and PI3K pathway. Endothelium-intact rings were incubated with cumulative doses of SF in the absence (triangles) or presence of PI3K inhibitors: (**A**) Wortmannin (0.1 µM; squares) or (**B**) LY29400 (10 µM; squares). Data represent mean ± SEM (n = 5; *p* < 0.01 for Salvia versus Wortmannin + Salvia or LY29400 + Salvia). (**C**) Modulation of Akt phosphorylation by SF. Aortic rings were incubated in either the absence (veh) or presence of wortmannin (wort) for 30 minutes. Rings were then pre-contracted with NE (3 µM) followed by treatment with SF (0.67 mg/ml) for 15 minutes. Proteins were extracted and subjected to SDS-PAGE against phosphorylated and total Akt levels. Bar graph represents normalization of the phosphorylated Akt levels to total ones. Bars with similar letters are significantly different; *p* < 0.05); n = 4 rings. (**D**) Rings were incubated with or without SF (0.67 mg/ml) after having been pre-treated without (vehicle) or with wortmannin (wort; ﻿0.1 µM) for 30 minutes. Then, cGMP levels were determined. n = 5; *denotes a *p* < 0.05.

The role of PI3K-Akt signaling axis in endothelium-dependent aortic relaxation is well-established^41, 42^. To determine if this signaling cascade is involved in the SF-induced relaxation, we utilized two structurally incongruent inhibitors of PI3K, wortmannin and LY294002. Indeed, pretreatment with either inhibitor caused a significant decrease in SF-induced vasorelaxation, with the maximum relaxation mitigated with no effect on potency. In control rings, pED_50_ was 4.72 ± 0.08 g/ml with 95% confidence interval of 4.89–4.55 g/ml. In wortmannin-preincubated rings, pED_50_ was 4.29 ± 0.34 with 95% confidence interval of 4.98–3.60 g/ml. On the other hand, R_max_ was 90.0 ± 4.3% for wortmannin-independent relaxation and 49.1 ± 11.5% in the presence of wortmannin (*p* < 0.01, Fig. 6A). Similar results were obtained with LY294002 (10 µM) pre-treatment (*p* < 0.01, Fig. 6B). In LY294002-preincubated rings, pED_50_ was 4.36 ± 0.13 with 95% confidence interval of 4.64–4.08 g/ml, and R_max_ was 77.4 ± 7.5% for wortmannin-independent relaxation and 44.5 ± 2.4% in the presence of LY294002.

To further confirm the participation of Akt in the SF-induced vasorelaxant pathway, we determined the level of Akt phosphorylation in SF-treated rings that were pre-incubated with or without wortmannin. Indeed, SF evoked significant increase in phosphorylation of Akt which was significantly inhibited by wortmannin (n = 3; *p* < 0.05) (Fig. 6C). Furthermore, pre-treatment with tricirbine (10 µM), which selectively inhibits Akt but not PI3K, abolished the SF-induced relaxation (data not shown). Taken together, the above data clearly implicates activation of the PI3-K/Akt signaling axis in SF-induced aortic relaxation.

To further validate the role of PI3K/Akt cascade in SF-induced relaxation, we pre-incubated rings with wortmannin followed by SF treatment and then we assessed cGMP levels. In line with the above results, pre-treatment with wortmannin (0.1 µM) significantly abolished SF-induced cGMP accumulation (Fig. 6D)

### Role of Potassium Channels in SF-induced relaxation of aortic rings

**Figure 7:**
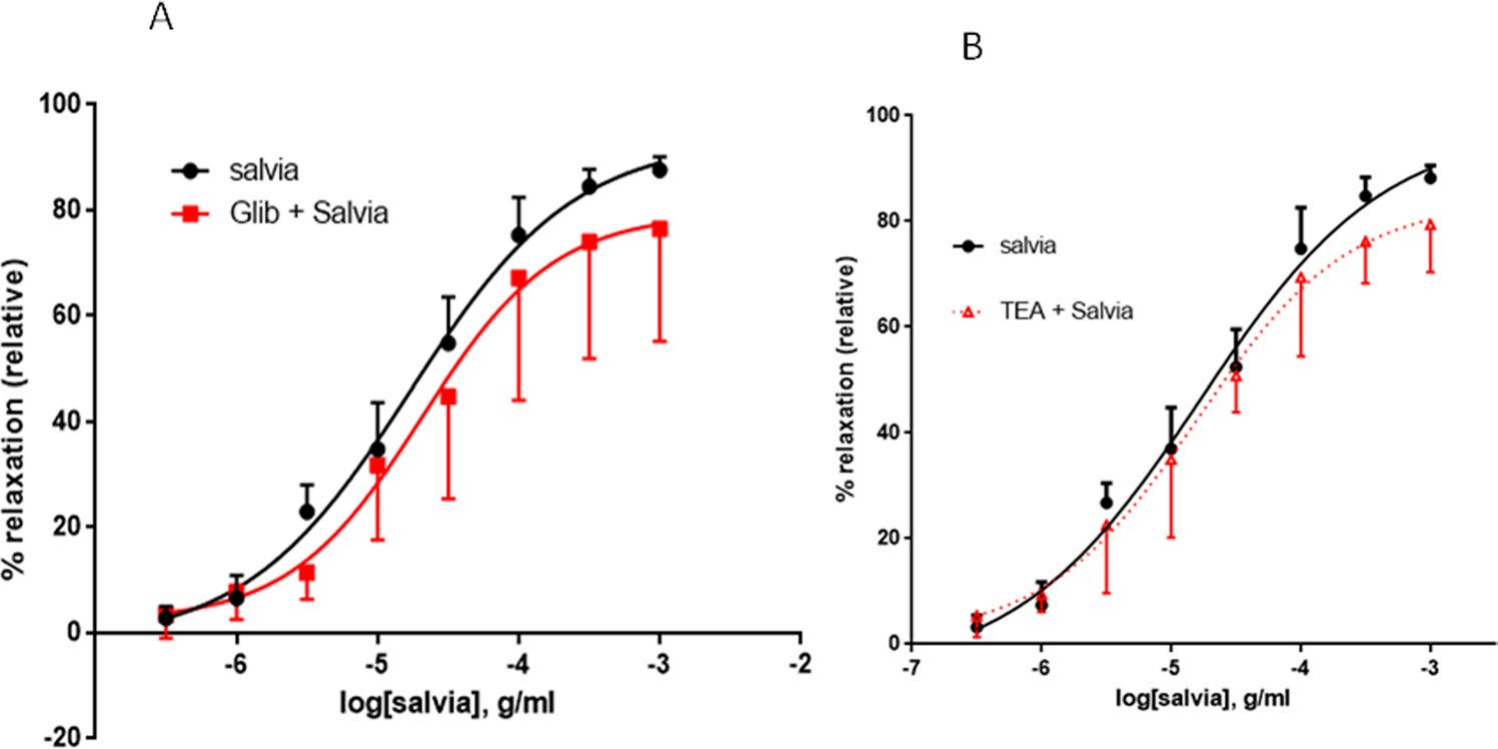
Role of potassium channels in SF-induced relaxation of aortic rings. Endothelium-intact rings were incubated with cumulative doses of SF in the absence (Salvia plus vehicle; filled circles) or presence of (**A**) 10 µM of Glibenclamide (Glib + Salvia; squares), or (**B**) Tetraethylammonium (100 µM) (TEA + Salvia; triangles). n = 6 or 5 for glibenclamide or TEA experiments, respectively. In both cases, no difference was noted between inhibitor treated and vehicle administered curves; *p* > 0.05 for Salvia alone versus either Glib + Salvia or Salvia + TEA.

Increase in the bioavailability of NO is known to cause vasorelaxation via the activation of calcium-dependent potassium channels^43^. Interestingly, prior inhibition of ATP-dependent or calcium-activated potassium channels caused no effect on relaxant responses to SF. Indeed, in vehicle-treated rings, pED_50_ was 4.77 ± 0.07 g/ml with 95% confidence interval of 4.91–4.63 g/ml. Comparable results were obtained with the same rings pre-treated with glibenclamide, an ATP-sensitive potassium channels blocker, where pED_50_ was 4.69 ± 0.17 with 95% confidence interval of 5.03–4.34 g/ml. Similarly, R_max_ was 93.4 ± 3.8% for inhibitor-independent relaxation and 79.4 ± 7.7% relaxation in the presence of glibenclamide (*p* > 0.05) (Fig. 7A). Similar results were obtained when Ca^++^-activated potassium channels were inhibited with tetraethylammonium, TEA (Fig. 7B). In vehicle-treated rings, pED_50_ was 4.78 ± 0.1 with 95% confidence interval of 4.98–4.58 g/ml, whereas TEA-pretreatment of the rings produced a pED_50_ value of 4.80 ± 0.15 with 95% confidence interval of 5.10–4.49 g/ml. Similarly, R_max_ was 97.9 ± 5.9% for inhibitor-independent relaxation and 84.06 ± 6.8% relaxation in the presence of TEA (*p* > 0.05) (Fig. 7B).

### Involvement of calcium channels

**Figure 8:**
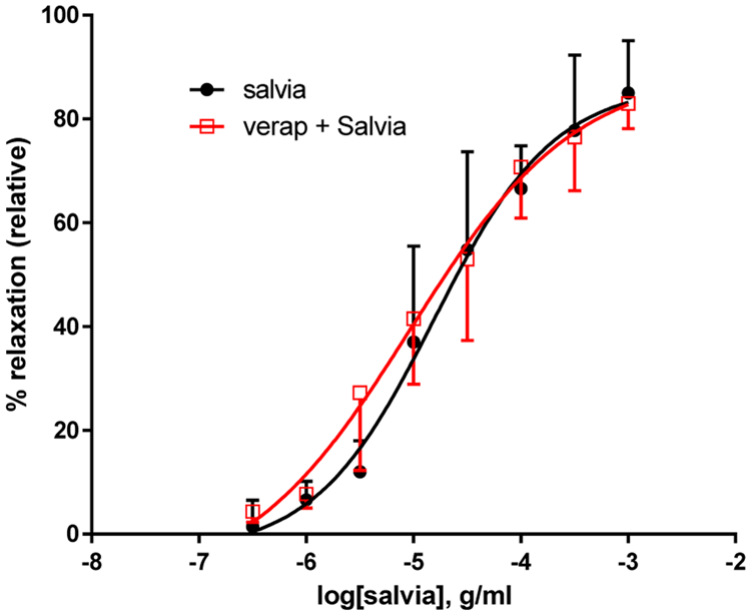
Role of Calcium Channels in SF-induced relaxation of aortic rings. Endothelium-intact rings were incubated with cumulative doses of SF in the absence (Salvia; circles) or presence of verapamil (1 µM; verap + Salvia; squares). n = 5; *p > *0.05.

To establish if calcium channels participate in SF-induced relaxation, we exposed the aortic rings to verapamil, a L-type calcium channel blocker, and there was no significant difference between the control and treated vessels (*p* > 0.05) (Fig. 8). In fact, results in vehicle-treated rings (pED_50_ was 4.81 ± 0.15 g/ml with 95% confidence interval of 5.11–4.50 g/ml) were similar to verapamil-incubated rings (pED_50_ was 5.03 ± 0.28 with 95% confidence interval of 5.61–4.46 g/ml). Likewise, R_max_ was 86.6 ± 7.4% or 89.5 ± 11.5% in the absence or presence of verapamil, respectively (Fig. 8).

### Involvement of cyclooxygenases

**Figure 9:**
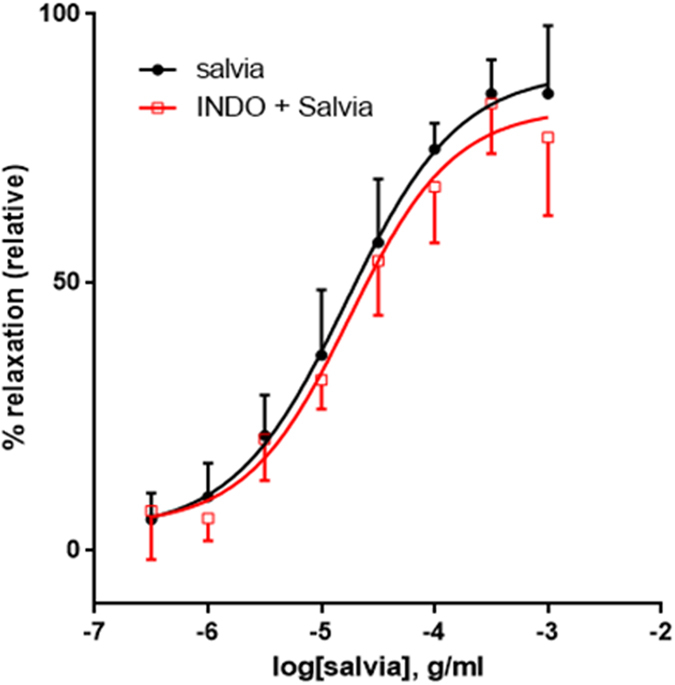
Role of cyclooxygenases in SF-induced relaxation of aortic rings. Endothelium-intact rings were incubated with cumulative doses of SF in the absence (Salvia; circles) or presence of indomethacin (10 µM; Indo + Salvia; squares). Data represent mean ± SEM (n = 5; *p > *0.05).

Prostanoids are known to regulate vascular tone^44, 45^. Here, we show that indomethacin, a non-selective cyclooxygenase blocker, did not significantly alter relaxant responses of rat aortic rings to SF (*p* > 0.05). Indeed, in salvia-treated rings, pED_50_ was 4.78 ± 0.10 g/ml with 95% confidence interval: 4.98–4.58 g/ml. And in indomethacin-treated rings, pED_50_ was 4.75 ± 0.11 g/ml with 95% confidence interval of 4.97–4.53 g/ml. Similarly R_max_ is 89.3 ± 4.6% for untreated relaxation and 82.4 ± 4.7% for indomethacin-exposed relaxation (Fig. 9).

### Involvement of histaminergic or muscarinic receptors

**Figure 10:**
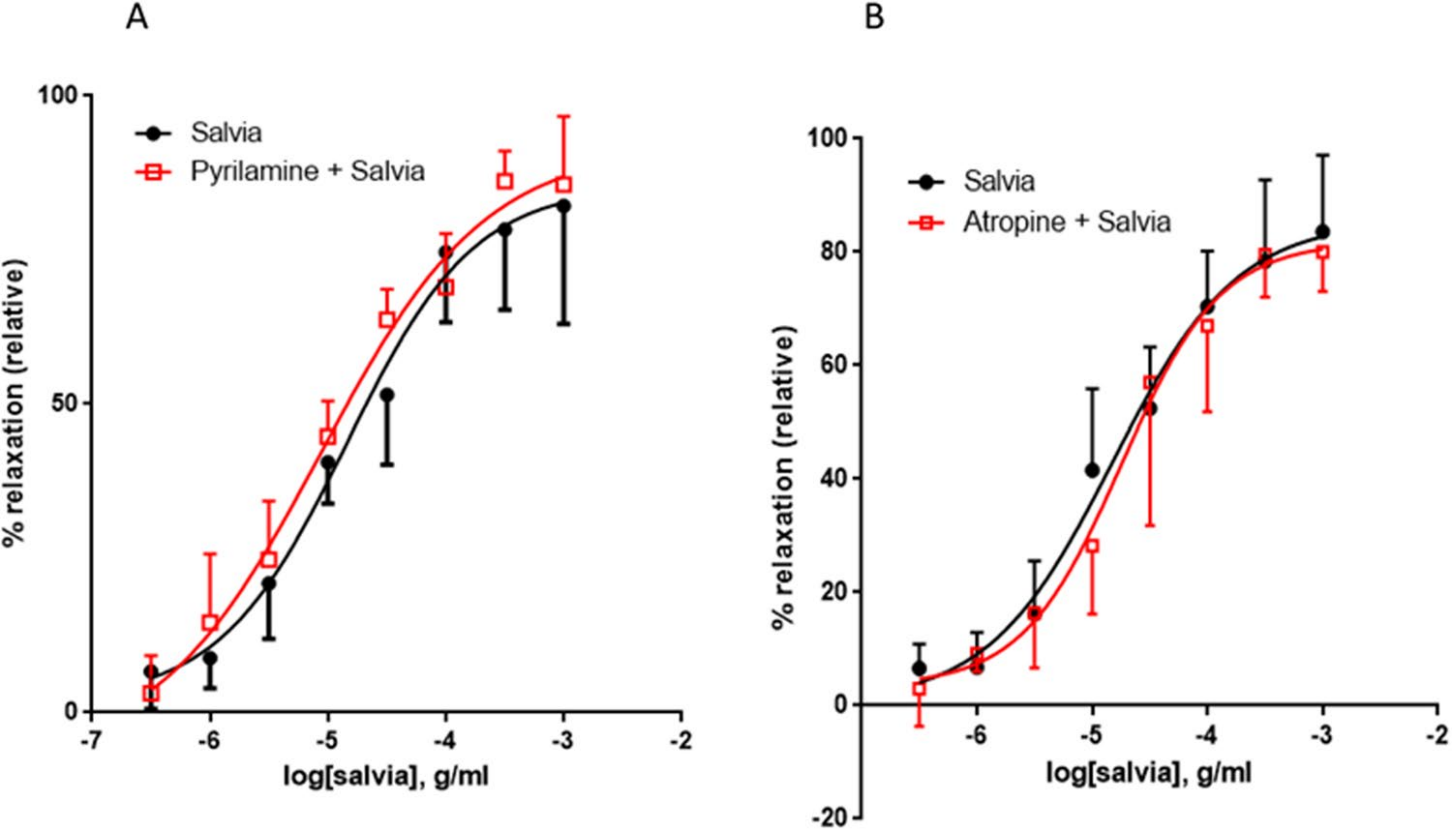
Involvement of histaminergic or muscarinic receptors in SF-induced vasorelaxation. Endothelium-intact rings were exposed to cumulative doses of SF in the absence (Salvia; circles) or presence of (**A**) 10 µM of pyrilamine (pyrilamine + Salvia; squares), or (**B**) 10 µM of atropine (atropine + Salvia; squares). Data represent mean ± SEM (n = 6 or 5 for pyrilamine or atropine experiments, respectively). *p* > 0.05 for Salvia alone versus either pyrilamine + Salvia or Salvia + atropine.

Penultimately, we investigated the influence of pyrilamine, a blocker of histamine H1-receptors on SF-generated aortic relaxation. In rings treated with salvia alone, pED_50_ was 4.84 ± 0.13 g/ml with 95% confidence interval of 5.11–4.57 g/ml. These results are analogous to those obtained in the presence of pyrilamine, where pED_50_ was 5.06 ± 0.15 with 95% confidence interval of 5.36–4.76 g/ml, to increasing doses of SF-mediated relaxation. Similarly, R_max_ is 85 ± 6% for rings treated with salvia alone, versus92 ± 6% for pyrilamine exposed ones, Fig. 10A). Pyrilamine did not have a significant effect on SF-induced relaxation (*p* > 0.05).

Lastly, we also determined the effect of atropine, a natural alkaloid that acts as a non-selective blocker of muscarinic receptors. In control rings, pED_50_ value was 4.82 ± 0.14 g/ml with 95% confidence interval of 5.11–4.54 g/ml. By comparison, in atropine-treated rings, pED_50_ was 4.75 ± 0.13 with 95% confidence interval of 5.01–4.49 g/ml to increasing doses of SF-mediated relaxation. Similarly, R_max_ is 86 ± 6% for inhibitor-independent relaxation and 82 ± 6% for atropine-treated relaxation, and magnitude of maximal relaxation is similar in both groups, Fig. 10B). Therefore, atropine does not have any significant effect on SF-induced relaxation (*p* > 0.05).

## Discussion

**Figure 11:**
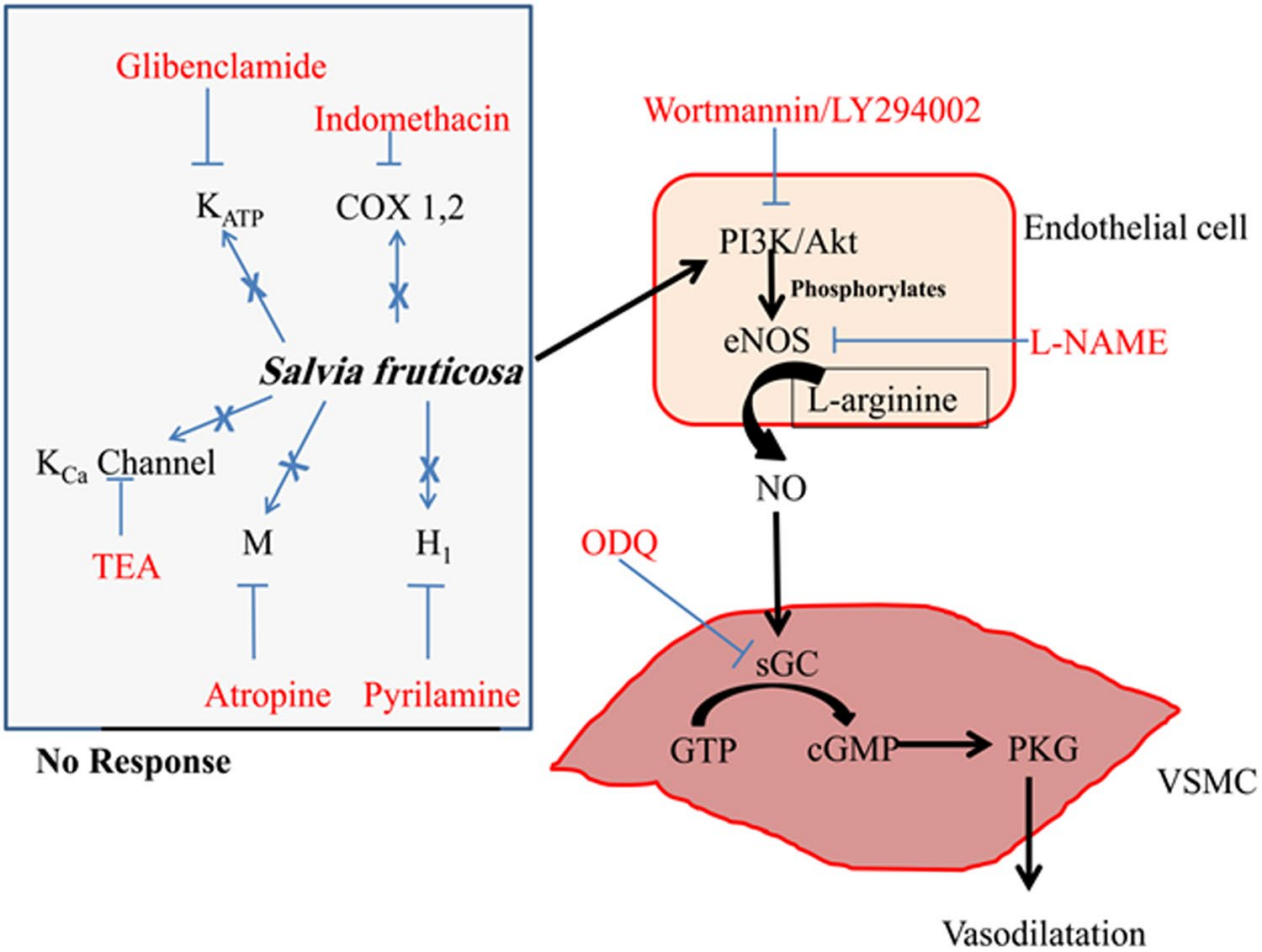
Schematic representation of the mechanism of *Salvia fruticosa*-induced relaxation of rat isolated aorta. The diagram displays the central role of nitric oxide, and its upstream activators and downstream effectors. Pharmacologic inhibitors are shown in red font color. Blue crosses reveal no involvement in the mechanism of SF-induced relaxation. The pathways for SF mechanism of action are drawn in black arrows.

The objective of the current experiments was to determine the pharmacological effects of ethanolic extract of *Salvia fruticosa* Mill. leaves on rat isolated thoracic aorta. From these observations, the salient feature of our investigation points to the key role of the PI3K/Akt, endothelial nitric oxide synthase, nitric oxide, soluble guanylyl cyclase and cGMP pathway (Fig. 11). To our knowledge, this is the first investigation to examine the detailed effects of extracts from any Salvia species activating the PI3K through to cGMP pathway. Additionally,our results show that the dose-dependent relaxation responses to *S. fruticosa* Mill. are contingent on endothelial cells. This is reflected by the observations that relaxation was significantly diminished both upon denudation of luminal endothelial layer and by inhibition of eNOS by L-NAME.

In native, healthy endothelia, there is a choreographed balance between the actions of vasoconstrictors (pro-inflammatory) and vasodilators (anti-inflammatory). When this equilibrium is shifted, a derangement in vascular homeostasis ensues. The vascular dysfunction of the arterial tree is a prelude to hypertension. Ample evidence exists to support the notion of vascular abnormalities (functional, mechanical and structural – vascular remodeling) in hypertension affecting not only small vessels, but also large calibre arteries^5, 6, 46–48^. Moreover, the homeostatic disturbance results in augmentation of vascular tone, activation of inflammatory responses, proliferation of VSMCs, and eventually precipitates an atherothrombogenic milieu^9, 49, 50^. Hence, a healthy life-style, embracing the intake of protective nutrients, such as *S. fruticosa* Mill., is imperative to ensure that endothelial cell homeostasis is optimally maintained.

The endothelium plays a critical role in determining vasotone, and receptors located on the endothelial surface are primary targets for initiating vasodilatory effects^51^ Activation of muscarinic M3 subtype^52^ and histaminergic H1^53^ receptors will induce relaxation of blood vessels. From our observations, we can conclude that SF–induced aortic relaxation is not mediated by muscarinic or histaminergic receptors. Accordingly, these results are consistent with a previous work showing their lack of involvement in relaxant effect of Danshen on rat isolated femoral arteries^54^.

Stimulation of the PI3K/Akt signaling pathway axis triggers a network of multiple outcomes. Accumulated evidence shows PI3K phosphorylates Akt at Ser473 causing the kinase to be activated, and to mediate downstream signaling processes associated with cell growth and migration^44, 55–58^, but has also been implicated in endothelium-dependent relaxation^41, 42^. More pertinently, eNOS has been found to be activated via the PI3K/Akt pathway by natural compounds like epigallocatechin-3-gallate^59^. Recently, in an ischemia/reperfusion injury model, it was found that ginsenosides, the major bioactive components of species panax ginseng, increase coronary arterial flow through stimulation of PI3K/Akt/eNOS mechanistic route. This demonstrates that the application of isolated extracts of ginseng bestowed protection against myocardial damage^60^. Similarly, in another study, activation of PI3K/Akt/NO pathway by the principle metabolite of ginsenosides evoked cardioprotection in myocardial ischemia-reperfusion injury^61^.

Stimulation of Akt enhances the activity of eNOS by phosphorylating serine at 1177^62, 63^. This, in turn, triggers increased release of nitric oxide (NO)^62–64^. NO then diffuses from the endothelial layer to the VSMCs wherein it activates the soluble guanylyl cyclase (sGC). Active sGC catalyzes the subsequent conversion of GTP to cGMP, which phosphorylates protein kinase G (PKG), a major intermediary in the vasorelaxation process^45^. Evidently, the results of our SF-induced aortic relaxation and dissection of its endothelium-reliant mechanistic route complements the aforementioned signaling mechanism through the PI3K/Akt/eNOS/NO/cGMP pathway. Notably, of all the Salvia species examined to date, this is the first study to show vasorelaxant effect from PI3K to cGMP in healthy normotensive vessels. In a corresponding manner, our results are in accord with a previous report demonstrating that extracts from the *species Salvia miltiorrhiza* (the dried root of which is commonly known as danshen) evoked endothelium-dependent relaxation that was reliant on NO and cGMP in rat aortic helical strips^21^. Conversely, endothelium-independent relaxation of rat coronary arteries responding to an aqueous extract of danshen has also been reported^65^.

Nitric oxide, a gaso-transmitter, is not only a potent vasodilator^66–68^, but also demonstrates antagonistic proliferative and thrombogenic behavior^69^. This endogenous intervention ensures vasculoprotection of the circulatory network by promoting homeostatic blood flow. In contrast, diminished NO bioavailability leads to a derangement in vasodilatory control, resulting in the onset and progression of hypertension as well as atherosclerosis among other vascular pathologies^49^.

Potassium channels play a critical role in regulating plasma membrane potential, and hence determining vascular tone^70, 71^. Increased bioavailability of nitric oxide is known to activate calcium-gated potassium channels^43^, which induce vasorelaxation by a mechanism involving hyperpolarization^72^. Our results suggest that neither Ca^2+^-dependent nor ATP-driven potassium channels are involved in SF-induced aortic relaxation. On the other hand, tanshinone IIA (a lipophilic diterpene quinone isolated from danshen) activates ATP-dependent potassium channels to decrease intracellular calcium concentration in rat aortic smooth muscle cells, and leading to relaxation^73^. Similarly, by using a 1000-fold higher concentration of TEA (100 mM) than that administered in the present study (100 µM), other researchers have demonstrated that danshen-induced participation of TEA-responsive potassium channels in endothelium-independent relaxation of rat coronary arteries^65^. These examples clearly illustrate the dichotomy in the members of genus Salvia, possibly due to differences in phytochemical profiles. Interestingly, ginsenoside-Re, a constituent of Panax *ginseng*, activates PI3K/Akt and NO signaling network to promote the opening of Ca^2+^-stimulated potassium channels in aortic smooth muscle cells^74^. The discrepancy that exists between our results and those outlined from the three different groups of investigators may be explained by highlighting the interplay of multiple contributory factors. Namely, disparity in Genus (Salvia versus Panax), dissimilarity in Saliva species (*fruticosa* versus *miltiorrhiza*), as well as the differences in parts of the plant (root versus leaves), each of these attributes will likely yield a highly variable phytochemical fingerprint. In this regard, some phytochemicals are exclusive to danshen (such as lipophilic tanshinones), whereas other constituents may share a commonality between the botanicals. An important point to take into account is that the concentration of each component will be highly variable due to location of harvesting, soil conditions, weather and time of harvest (season). In addition, there are methodological differences, for instance the applied concentration of antagonists (as is the case with TEA discussed above), extraction solvent and concentration of the herbal extract, and location of segment of vessel acquired from the arterial network (aorta versus coronary artery) will all contribute to disparity between the results.

Our data reveal that Ca^2+^ channels do not contribute to SF-induced relaxation of rat aortas. Interestingly, the blockage of calcium channels by danshen induces endothelium-independent relaxant activity in rat coronary arteries^65^. While this may appear to conflict with our study, it is important to note that different vascular beds behave differently even to the same stimulus^75, 76^. This apparent difference in response may also be explained by the points discussed above.

Cyclooxygenases (isoforms COX-1 and COX-2) are crucial enzymes in the conversion of arachidonic acid to biologically active prostanoids. This group of lipophilic compounds is known to play multiple roles in vascular biology, including vasodilation^67, 77^. One such potent vasodilator released by the endothelium is prostacyclin (PGI_2_), which acts via its corresponding G-protein coupled hepta-helical transmembrane domain receptors (IP)^78^. As a matter of fact, in the present investigation, the cyclooxygenase pathway does not appear to participate in SF-mediated relaxant activity. This concurs with an earlier report of a non-contributory function of prostaglandins to danshen-induced relaxant responses in rat femoral arteries^54^. This is not surprising as both herbs, danshen^79^ and SF^37^, are characterized by the presence of rosmarinic acid. More to the point, rosmarinic acid is a non-selective inhibitor of the enzyme isoforms COX-1 and COX-2^80^. Furthermore, the authors highlighted the property of inhibitory activity being comparable to the non-steroidal anti-inflammatory drugs (NSAIDs), aspirin, ibuprofen and naproxen^80^. Apparently, SF will be unable to recruit the prostanoid metabolites for regulation of vascular tone.

The molecular mechanism of action for SF in rat aorta is summarized in Fig. 11. Collectively, our investigation supports the health orientated benefits of SF as a vasodilator. All blood vessels contribute to the overall systemic resistance and consequent blood pressure. The aorta itself affects and is affected by changes in blood pressure. However, it is the resistance vessels that impart the biggest effect on vascular resistance. Therefore, the present studies are to be extended to resistance-size arteries (<300 µm internal diameter). If the results from these microvessels mirrored those obtained from aortic vessels, then this would confirm the potential for SF as a regulator for blood pressure. These studies may underscore and provide concrete confirmation for what we have observed in a large conduit vessel; and *raison d’etre* for its inclusion in ethnomedicine cabinet pertaining to inhabitants of the Levant. As such, these future investigations could substantiate the notion of *Salvia fruticosa* Mill. as an anti-hypertensive agent.

## Materials and Methods

### Preparation of the extract

Leaves of *Salvia fruticosa* Mill. were harvested from Yanouh area (East of Tyre, Lebanon) (GPS coordinates: 33°15′52 N and 35°17′53 E). No specific permission is required for collecting this plant or for using the land where it is located. This plant is neither an endangered nor a protected species; it is readily and commercially available in the market.

The SF extract was prepared as in our recent publications of two other herbs^16, 81, 82^. Leaves were rinsed in distilled water to remove any dust or dirt and air-dried in the dark at room temperature. After grinding the leaves with a mortar and pestle, the powder was extracted three times in 70% aqueous ethanol and the mixture kept in the dark for 72 hours at 4 degrees without stirring. The mixture was then filtered through a sintered glass funnel and the filtrate evaporated to dryness using rotary evaporator at room temperature. The obtained residue was collected and kept at −20 °C until further use, when it would be dissolved in aqueous ethanol.

### Preparation of rat isolated thoracic aortae

#### Ethics Statement

All procedures used within the present study were performed in strict compliance with the recommendations in the Guide for the Care and Use of Laboratory Animals of the National Institutes of Health. Institutional approval was obtained for all protocols and procedures (Scientific Committee in the Faculty of Public Health in the Lebanese University; (Permit number for Samaha Salvia Project: UL/FSPIV/07/2011)). All efforts were made to minimize suffering.

### Animals

Experiments in this study were conducted on male Sprague-Dawley rats weighing 230–290 gm. Animals were kept under a 12:12 hours light: dark cycle at a temperature of 23 ± 2 °C and had access to food and water *ad libitum*.

### Drugs and Chemicals

Norepinephrine, 3-isobutyl-1-methylxanthine (IBMX), acetylcholine, Nω-nitro-L-arginine methyl ester (L-NAME), atropine, pyrilamine, verapamil, indomethacin, tetraethylammonium (TEA), 1H-[1,2,4]oxadiazolo[4,3-alpha]quinoxalin-1-one (ODQ) were purchased from Sigma-Aldrich Co. (St. Louis, MO, USA). Wortmannin and LY294002 were obtained from Alexis Biochemicals (USA) and Tocris (USA), respectively.

### Quantification of NO production

As we recently described^82^, NO production was determined by calculating the amount of nitrate/nitrite produced based on the Griess reaction. A colorimetric assay kit (Cayman Chemicals, Michigan, USA) was used as per the manufacturer’s instructions^83^. Briefly aortic homogenates and Griess reagents were mixed and incubated at room temperature for 10 min and the absorbance was measured at 540 nm wavelength. The amount of nitrite formed was normalized to the protein content of the respective aortic rings. A standard curve was constructed using known concentrations of sodium nitrite. The absorbance value of blank wells were subtracted from the values of the wells with the rings.

### Preparation of Aortic Rings and Tension Measurement

On the day of experimentation, the animals were euthanized by an overdose of phenobarbital (50 mg/kg body weight) and the thoracic aorta was rapidly dissected and isolated, and placed in cold (4 °C) modified Krebs-Henseleit buffer (KHB) pre-equilibrated with 5% CO_2_ in oxygen. The aorta was carefully cleaned of adhering connective and fat tissues, and cut into ring segments of 2 to 3 mm in length. Each aortic ring was suspended by a pair of stainless steel hook (stationary) and stirrup (attached to isometric force transducer) in a pre-warmed (37 ± 2 °C) organ bath chamber containing a modified Krebs-Henseleit buffer (KHB, pH 7.4, 37 ± 2 °C) with the composition previously described^84^ (mM): NaCl (130), Mg_2_SO_4_ *·*7H_2_O (1.17), NaHCO_3_ (14.9), KCl (4.7), KH_2_PO_4_ (1.18), CaCl_2_ *·*2H_2_O (1.6) and glucose (5.5). This solution was aerated with 95% O_2_ and 5% CO_2_ to maintain the pH of KHB at 7.4. During the initial equilibration period of 1 hr, the rings were subjected to isometric resting tension of 2 g in a step-wise manner as previously described^85^. Aortic ring tension responses were recorded by a computerized data acquisition system (AD Instruments, UK) and Chart software (AD Instruments, UK). Subsequently, the vessel integrity was determined by application of 3 µM NE, followed by 2 exposures to 80 mM KCl, with thorough washes with KHB in between each activation of the aorta. If the tension recordings to the two KCl challenges were similar, the protocols below were followed.

### Experimental protocols

Endothelium-intact aortic rings were precontracted with norepinephrine (NE) (3 µM). Cumulative concentrations (0.3 µg/ml to 1 mg/ml, at half-log doses) of SF were added to determine the rings’ reactivity. At each dose of SF, the curve was allowed to reach a plateau before the addition of subsequent dose. To ensure that integrity of the endothelium was maintained, vascular relaxant response to Ach (10 µM) was assessed in each vessel following precontraction with NE (>80% relaxation of NE-dependent contraction). When the involvement of the endothelium in the observed relaxation was desired, vessels were denuded of the endothelial layer. Failure of Ach to produce a vasorelaxant response greater than 25% was assumed as an indication of effective endothelium denudation. Cumulative dose response curves (0.3 µg/ml to 1 mg/ml, at half-log doses) of SF were then determined in these endothelial-stripped rings.

The concentrations of inhibitors/antagonists used in the proceeding experiments have already been described previously^86^, each blocker was incubated for 30 min as appropriate, and followed SF’s ability to relax aortic segments. The solvents used for dissolving the blockers are indicated below (following each inhibitor). None of the vehicles/solvents used has any vasorelaxing effects on its own at all the doses used.

To determine the role of NO, rings with intact endothelium were pretreated with L-NAME (100 *μ*M), a nitric oxide (NO) synthase inhibitor, for 30 minutes. Then, aortic rings were contracted with NE, followed by treatment with SF.

To investigate the role of guanylate cyclase, prostaglandins, potassium channels, muscarinic receptors, histamine H1-receptors or calcium channels in SF-induced relaxation, the following inhibitors were used: oxadiazole quinoxalin (ODQ, 10 µM), a soluble guanylate cyclase inhibitor; Indomethacin (10 µM), a non-selective cyclooxygenase inhibitor; Glibenclamide (10 µM), an ATP-sensitive potassium channels blocker, or Tetraethylammonium (100 µM), a non-selective inhibitor of Ca^++^-activated potassium channels; pyrilamine (10 μM), a histamine H1-receptor antagonist or atropine (10 μM), a muscarinic receptor antagonist; and Verapamil hydrochloride (1 µM), a L-type Calcium channel blocker.

The role of the PI3K/Akt signaling was assessed by pre-incubating the rings with wortmannin (100 nM) or LY294002 (10 µM) for 30 minutes. This was followed by cumulative dose response curve for treatments with SF.

To validate that no significant irreversible or residual effect on vascular responsiveness to SF extract has taken place, the aortic rings that had been exposed to cumulative concentrations of SF were washed for 45 to 60 minutes, followed by new exposure to NE (3 *μ*M) and Ach (10 *μ*M). Finally, the rings were challenged again with KCl (80 mM) to confirm the viability of the vessels.

### cGMP assay

Pre-equilibrated rings were incubated in 3-isobutyl-1-methylxanthine (IBMX, a competitive nonselective phosphodiesterase inhibitor; 0.1 mM) for 30 minutes before addition of NE. Vessels were then allowed to equilibrate for an additional 30 minutes before addition of SF. The reaction was then quickly stopped by freezing the tissues in liquid nitrogen. After homogenization in trichloroacetic acid, samples were centrifuged at 10000 × g for 10 min and the supernatant was extracted five times with water-saturated diethylether. The cGMP and protein content in the extract were determined by a specific immunoassay (Amersham Biosciences (now GE Healthcare Life Sciences), USA) and by the use of Bradford’s method, respectively. Results are expressed as pmoles of cGMP per milligram of protein.

### Western blotting

Rings were homogenized in a sample buffer containing 2% SDS and 60 mM Tris ⋅ HCl (pH = 6.8) at room temperature. After clearing the homogenate by centrifugation (14,000 *g*, 10 min, 4 °C), the supernatant was collected. Protein concentrations were determined using the bincinchoninic acid assay (Pierce). Equal amounts of proteins were loaded and resolved on a 10% sodium dodecyl sulfate-polyacrylamide gel electrophoresis (SDS-PAGE) and transferred to polyvinyldifluoride membrane (Millipore). After blocking with 10% nonfat dry milk in Tris-buffered saline containing 0.1% Tween 20, membranes were washed then incubated with primary antibodies for total or phosphorylated Akt (Cell Signaling, USA) at 4 °C, overnight. The membrane was then washed three times and incubated with HRP-conjugated secondary antibody for 1 hour. After extensive washings, blots were developed using enhanced chemiluminescence (Amersham Biosciences, USA).

### Statistical analysis

Data are presented as means ± SEM. Relaxations to SF are expressed as a percentage decrease of the NE contraction. Each cumulative dose-effect curve for SF-induced relaxation was plotted, contingent on application of sigmoidal curve fitting and non-linear regression, using Prism version 6 (GraphPad Software Inc., San Diego, CA., U.S.A.) to generate the parameters R_max_ (maximal relaxant response) and pED_50_ (the negative logarithm of SF dose required to give 50% of the maximum aortic-relaxant response, R_max_). Statistical analysis of the data was performed by Student’s *t*-test. When more than two means were compared, Analysis of Variance (ANOVA) was used: either a one-way ANOVA with Tukey’s post hoc test (data represented as bar graphs) or a two-way ANOVA with Sidak’s multiple comparison post hoc test (data represented as sigmoidal curves) (Prism, GraphPad Software, San Diego, CA). A *p* value of less than 0.05 was considered statistically significant.
